# Unsupervised outlier detection applied to SARS-CoV-2 nucleotide sequences can identify sequences of common variants and other variants of interest

**DOI:** 10.1186/s12859-022-05105-y

**Published:** 2022-12-19

**Authors:** Georg Hahn, Sanghun Lee, Dmitry Prokopenko, Jonathan Abraham, Tanya Novak, Julian Hecker, Michael Cho, Surender Khurana, Lindsey R. Baden, Adrienne G. Randolph, Scott T. Weiss, Christoph Lange

**Affiliations:** 1 Department of Biostatistics, T.H. Chan School of Public Health, Harvard University, Boston, MA 02115 USA; 2 Department of Medical Consilience, Graduate School, Dankook University, Yongin, South Korea; 3 Genetics and Aging Research Unit, Department of Neurology, McCance Center for Brain Health, Massachusetts General Hospital, Boston, MA 02114 USA; 4 Department of Microbiology, Harvard Medical School, Blavatnik Institute, 77 Avenue Louis Pasteur, Boston, MA 02115 USA; 5 Department of Anesthesiology, Critical Care and Pain Medicine, Boston Children’s Hospital, Boston, MA 02115 USA; 6 Harvard Medical School, Harvard University, Boston, MA 02115 USA; 7 Channing Division of Network Medicine, Department of Medicine, Brigham and Women’s Hospital, Boston, MA 02115 USA; 8 Food and Drug Administration, Silver Spring, MD 20993 USA; 9 Division of Infectious Diseases, Harvard Medical School, Brigham and Women’s Hospital, Boston, MA 02115 USA

**Keywords:** SARS-CoV-2, Nucleotide sequences, Outlier detection, Variants of interest, Machine learning

## Abstract

As of June 2022, the GISAID database contains more than 11 million SARS-CoV-2 genomes, including several thousand nucleotide sequences for the most common variants such as delta or omicron. These SARS-CoV-2 strains have been collected from patients around the world since the beginning of the pandemic. We start by assessing the similarity of all pairs of nucleotide sequences using the Jaccard index and principal component analysis. As shown previously in the literature, an unsupervised cluster analysis applied to the SARS-CoV-2 genomes results in clusters of sequences according to certain characteristics such as their strain or their clade. Importantly, we observe that nucleotide sequences of common variants are often outliers in clusters of sequences stemming from variants identified earlier on during the pandemic. Motivated by this finding, we are interested in applying outlier detection to nucleotide sequences. We demonstrate that nucleotide sequences of common variants (such as alpha, delta, or omicron) can be identified solely based on a statistical outlier criterion. We argue that outlier detection might be a useful surveillance tool to identify emerging variants in real time as the pandemic progresses.

**Supplementary Information:**

The online version contains supplementary material available at 10.1186/s12859-022-05105-y.

## Introduction

More than 13 million nucleotide sequences of the SARS-CoV-2 virus have been collected from patients around the world since the beginning of the pandemic and made available in the GISAID database [1, 2]. Among them are thousands of nucleotide sequences of the most common variants, precisely for the alpha (B.1.1.7), beta (B.1.351), delta (B.1.617.2), gamma (P.1), GH (B.1.640), lambda (C.37), mu (B.1.621), and omicron (B.1.1.529) variants [3].

The emergence of new variants of the SARS-CoV-2 virus poses a threat to the progress made by ongoing vaccination campaigns against COVID-19. Therefore, the detection and possible identification of newly emerging variants of the SARS-CoV-2 virus in (close to) real time is of great interest.

Currently, a tool called “genomic surveillance” is used by the Centers for Disease Control (CDC) to detect new variants [4]. This is done both through the National SARS-CoV-2 Strain Surveillance (NS3) program, as well as through commercial and academic laboratories contracted by the CDC, where genetic information of SARS-CoV-2 specimen are analyzed and classified into variants. By definition, a variant is characterized by having one or more mutations which differentiate it from other variants of the SARS-CoV-2 virus [5]. A group of variants with similar genetic changes (a lineage) can be classified as a variant of concern (VOC) or a variant of interest (VOI) if they share characteristics that potentially necessitate public health action. For example, the U.S. government SARS-CoV-2 Interagency Group (SIG) classified omicron as a Variant of Concern (VOC) on 30 November 2021 due to the fact that omicron emerged in multiple countries without apparent travel history, the replacement of certain delta variants as predominant variants in South Africa by omicron, and its number of mutations in the spike protein which indicated a reduced susceptibility to sera from vaccinated individuals and certain monoclonal antibody treatments. The purpose of this article is to explore the ability of new unsupervised learning methodology to detect emerging variants of interest.

As shown previously in the literature [6, 7], an unsupervised cluster analysis in which the similarity of all pairs of nucleotide sequences is assessed using the Jaccard index, and subsequent application of principal component analysis to the Jaccard similarity matrix, results in clusters of sequences according to certain characteristics such as their strain or their clade. Importantly, in [8] the authors notice that nucleotide sequences the omicron variant cluster among sequences stemming from variants identified earlier on during the pandemic. Due to the fact that the aforementioned unsupervised approaches successfully clustered nucleotide sequences by strain or clade, and revealed features of the omicron variant, we likewise focus on an unsupervised approach based on the Jaccard similarity matrix in connection with principal component analysis in this work.

This finding immediately prompts the question whether the nucleotide sequences belonging to common variants can be identified by unsupervised outlier detection. In this article, we investigate this question by applying outlier detection to nucleotide sequences, both before the emergence of a variant and after a variant has emerged. We demonstrate that indeed, the number of detected outliers often increases shortly after the emergence of a new variant, and that nucleotide sequences of common variants can be identified solely based on a statistical outlier criterion.

Our findings could have important implications for the automated, unsupervised identifications of SARS-CoV-2 strains. We argue that outlier detection might be a useful surveillance tool to identify emerging variants of interest in real time as the pandemic progresses. This is also important for vaccination strategies, to identify emerging variants that may be resistant to available vaccines [9].

The article is structured as follows. The “Methods” section introduces the methodology we use for this article, starting with data acquisition and cleaning, and how the similarity of sequences is assessed. We then describe the outlier detection method we use. The “Results” section presents our findings on the clustering and outlier detection of SARS-CoV-2 nucleotide sequences. The article concludes with a “Discussion” section.

## Methods

In this section, we highlight methodological features of the analysis. In particular, we describe data acquisition and cleaning (“Data acquisition and cleaning” section), the assessment of the similarity of nucleotide sequences (“Assessing the similarity of nucleotide sequences” section), the methods used for outlier detection among sequences (“Outlier detection” section), and the calibration of the outlier detection (“Calibration” section).

### Data acquisition and cleaning

All findings reported in this article are based on an image of all available SARS-CoV-2 nucleotide sequences in the GISAID database [1, 2] until 28 March 2022, consisting of 211,167 sequences having accession numbers in the range of EPI_ISL_403962–EPI_ISL_11498019. By timestamp we always refer to the collection date on GISAID. Sequences are only included in the analysis if they satisfy the four data quality attributes on GISAID. To be precise, all nucleotide sequences have to satisfy the criterion of being *complete* (defined as sequences having length at least 29,000 bp), *high coverage* (defined as sequences with less than 1% N-bases), *with patient status* (defined as sequences with meta information consisting of age, sex, and patient status), and *collection date complete* (defined as sequences with a complete year-month-day collection date) (Additional file 1).

We aim to investigate if it is possible to detect sequences of a new variant among the other sequences in circulation upon emergence of that new variant. We consider eight common SARS-CoV-2 variants available on GISAID. Those are alpha (B.1.1.7), beta (B.1.351), delta (B.1.617.2), gamma (P.1), GH (B.1.640), lambda (C.37), mu (B.1.621), and omicron (B.1.1.529) variants (Table 1).

**Table 1:** Local outlier detection approach

Variant	Before emergence of variant				After emergence of variant			
	T_1_	No. outliers	True positives	No. seq	T_2_	No. outliers	True positives	No. seq
Alpha	2020-10-01	1314	0	0	2021-02-16	1070	329	788
Beta	2020-02-18	78	0	0	2021-01-27	1902	88	99
Delta	2020-03-12	0	0	0	2021-07-21	212	175	1085
Gamma	2020-08-24	1589	0	0	2021-03-09	97	3	140
GH	2021-10-25	137	0	0	2021-11-22	179	0	4
Lambda	2021-01-17	2067	0	0	2021-01-18	2066	4	4
Mu	2021-03-07	0	0	0	2021-04-30	0	0	16
Omicron	2021-11-12	191	0	0	2021-12-26	276	19	25

Number of detected outliers in Figs. 4, 5, 6, 7, 8, 9, 10 and 11 before and after the emergence of each of the eight variants. True positives among the detected outliers, and number of sequences included for each variant

To detect a new variant, we generate two reference datasets for each variant. For the first dataset, we determine the timepoint T_1_ at which the first sequences of each variant under consideration emerge on GISAID. We then generate the first reference dataset using only sequences from GISAID with a timestamp before T_1_. The second dataset emulates the emergence of a new variant. For this we determine the timepoint T_2_ at which 10% of all the sequences of a variant under consideration are available on GISAID (the threshold of 10% is arbitrary). We then generate the second reference dataset using only sequences from GISAID with a timestamp up to T_2_. The details of the reference dataset up to T_1_ are given in Table 2, the sequences we aim to detect for each variant are given in Table 3, and the combined dataset simulating the emergence of each variant up to timepoint T_2_ is given in Table 4. As before, the timestamps T_1_ and T_2_ mentioned in the article and in Tables 2, 3 and 4 refer to the collection date on GISAID.

**Table 2:** Composition of the reference dataset

Variant	From accession ID	To accession ID	From date	To date	No. seq.
Alpha	403,963	11,229,661	2020-01-10	2020-09-30	9999
Beta	403,962	10,338,097	2020-01-08	2020-02-17	437
Delta	404,227	11,396,757	2020-01-10	2020-03-11	10,000
Gamma	403,962	11,448,682	2020-01-08	2020-08-23	9999
GH	408,430	11,468,153	2020-01-10	2021-10-24	10,000
Lambda	403,962	11,359,366	2020-01-08	2021-01-16	10,000
Mu	408,484	11,448,683	2020-01-10	2021-03-06	10,000
Omicron	412,970	11,468,160	2020-01-24	2021-11-11	10,000

The reference dataset is used as a baseline before the emergence of each new variant. Range of accession numbers extracted from the GISAID database, their time stamps, and the total number of sequences included

**Table 3:** Composition of the sequences (by variant) that we aim to detect, consisting of the first 10% of all sequences per variant

Variant	From accession ID	To accession ID	From date	To date	No. seq.
Alpha	733,573	11,230,479	2020-10-01	2021-02-15	788
Beta	660,611	10,980,370	2020-02-18	2021-01-26	99
Delta	1,716,736	11,267,911	2021-01-09	2021-07-20	1085
Gamma	875,689	11,396,742	2020-12-25	2021-03-08	140
GH	6,370,560	6,651,704	2021-11-03	2021-11-10	4
Lambda	1,111,316	1,111,334	2021-01-17	2021-01-17	4
Mu	2,500,943	5,196,329	2021-04-01	2021-04-29	16
Omicron	7,834,399	9,462,827	2021-12-04	2021-12-25	25

Range of accession numbers extracted from the GISAID database, their time stamps, and the total number of sequences included

**Table 4:** Combined dataset consisting of both Tables 2 and 3, subsampled again to size 10,000

Variant	From accession ID	To accession ID	From date	To date	No. seq.
Alpha	406,592	11,403,614	2020-01-08	2021-02-15	10,000
Beta	403,963	11,229,964	2020-01-10	2021-01-26	10,000
Delta	404,227	11,403,612	2020-01-16	2021-07-20	10,000
Gamma	407,079	11,448,683	2020-01-10	2021-03-08	10,000
GH	404,227	11,468,151	2020-01-10	2021-11-10	10,000
Lambda	406,593	11,330,894	2020-01-10	2021-01-17	10,000
Mu	408,489	11,468,147	2020-01-10	2021-04-29	10,000
Omicron	410,301	11,448,664	2020-01-13	2021-12-25	10,000

Range of accession numbers extracted from the GISAID database, their time stamps, and the total number of sequences included

Our planned subsequent computations on the nucleotide sequences (the calculation of the principal components of the Jaccard similarity matrix) are too computationally intensive to be carried out for all available sequences on GISAID. For this reason, we down-sample each dataset by drawing an unbiased sample of size 10,000 without replacement.

Using the alignment tool MAFFT [10] and the official SARS-CoV-2 reference sequence (available on GISAID under the accession number EPI_ISL_402124), we align all n sequences to the reference genome. We employed MAFFT with the *keeplength* option in order to obtain a well-defined window (of length L = 29,891 base pairs) for comparison of all sequences. All other parameters of MAFFT were kept at their default values.

### Assessing the similarity of nucleotide sequences

We next convert all sequences into a binary Hamming matrix X ∈ B^n x L^ (where B = {0,1} is the set of binary numbers) as follows. We compare the reference genome to each aligned nucleotide sequence, and set X_ij_ = 1 if the sequence with number i differs at position j from the reference sequence. Otherwise, we set X_ij_ = 0. Here, the number of rows of X is set to the number of nucleotide sequences, and L = 29,891 is the number of base pairs in the comparison window. The row sums of X correspond to the Hamming distance of each nucleotide sequence to the reference genome. This methodology has already been used in the literature [6–8, 11].

We employ the Jaccard similarity measure [12–14] to assess the similarity of all pairs of sequences. To be precise, each entry (i,j) of the Jaccard matrix J(X) ∈ ℝ^n x n^ (having n rows and n columns) is a measure of similarity between the binary rows i and j of X. An entry (i,j) of J(X) of zero encodes that the two genomes do not share any deviations from the reference genome, while an entry of one encodes equality of rows i and j of X. We employ the R-package “locStra”, available on CRAN [15, 16], to compute the Jaccard matrix.

For all figures included in this work, we visualize the Jaccard similarity measures by computing its first two principal components. We plot the first principal component against the second principal component, thus effectively interpreting the entries of the first eigenvector as x-coordinates, and the ones of the second eigenvector as y-coordinates. We color each point according to either a time stamp, according to its cluster membership, or according to whether it is an outlier.

### Outlier detection

We are interested in detecting sequences falling into neighborhoods or clusters in which they are classified as outliers (subject to a certain criterion). To be precise, we are interested in sequences falling into neighborhoods consisting of sequences having much older (or newer) time stamps.

We aim to utilize an approach which is not dependent on previously identified clusters. One way to achieve this is to define a local environment of radius eps > 0 around each sequence in a principal component plot (each sequence corresponds to a point in the principal component plot), and to consider all other (that is, similar) sequences falling into that local environment. Comparing the time stamp of the sequence under consideration to the distribution of timestamp in the local environment allows one to define an outlier. We say that a sequence is an outlier in its local environment if its time stamp is more than f > 0 standard deviations from the mean date in the environment.

### Calibration

Our clustering approach depends on two tuning parameters, the radius of the local environment eps, and the factor f that specifies how many standard deviations away from the mean date are needed to define a sequence as an outlier. To calibrate both parameters, we look at the number of outliers which are identified in the data as a function of both eps and f. This results in a typical “elbow” plot, though here in two dimensions (see Fig. 2). For small values of f, meaning values close to the mean, many outliers are flagged. As f increases, fewer and fewer outliers are identified. The decrease is usually not linear. Instead, the number of outliers usually drops rapidly at a certain cutoff f before leveling off, thus giving the plot its name. The point at which the plot levels off can be used to determine f. We apply the elbow method to both set the parameter f, as well as the parameter eps.

## Results

**Fig. 1:**
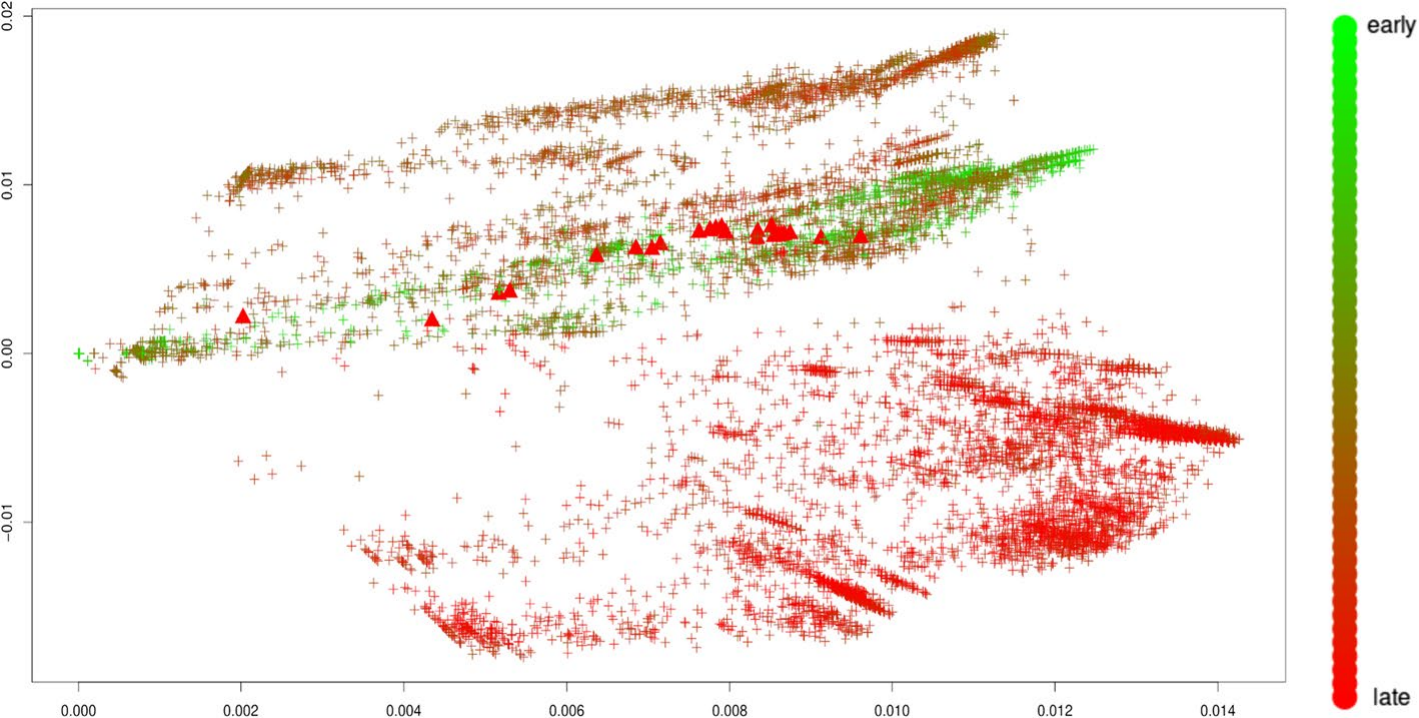
Omicron variant (see Table 4). First two principal components of the Jaccard matrix, colored by the collection time stamp of each nucleotide sequence. The color scale encodes early (green) to late (red) sequences according to the color scheme shown on the right. Sequences of the omicron variant (see Table 3) are highlighted as triangles

We first focus on the newest variant, omicron. Figure 1 shows a plot of the first two principal components of the Jaccard matrix as outlined in section “Assessing the similarity of nucleotide sequences”. As observed previously [8] the genomes from GISAID exhibit a particular progression pattern, with older sequences (green) clustering in the middle of the plot, while newer samples (red) cluster at the bottom of the plot. The progression of genomes seems to take place from the early point cloud (green, middle), to genomes with intermediate timestamps (top), to new samples (red, bottom). As also observed in the aforementioned publication, genomes of the omicron strain are most similar to genomes in stemming from early on in the pandemic. This is visible from Fig. 1 as omicron samples (triangles) fall into a point cloud of early (green) genomes.

Interestingly, the observations for Fig. 1 are virtually identical with the ones made in [8], even though both experiments are made with independent, and thus entirely different, subsamples without replacement of size 10,000 taken from all complete sequences available on GISAID.

**Fig. 2:**
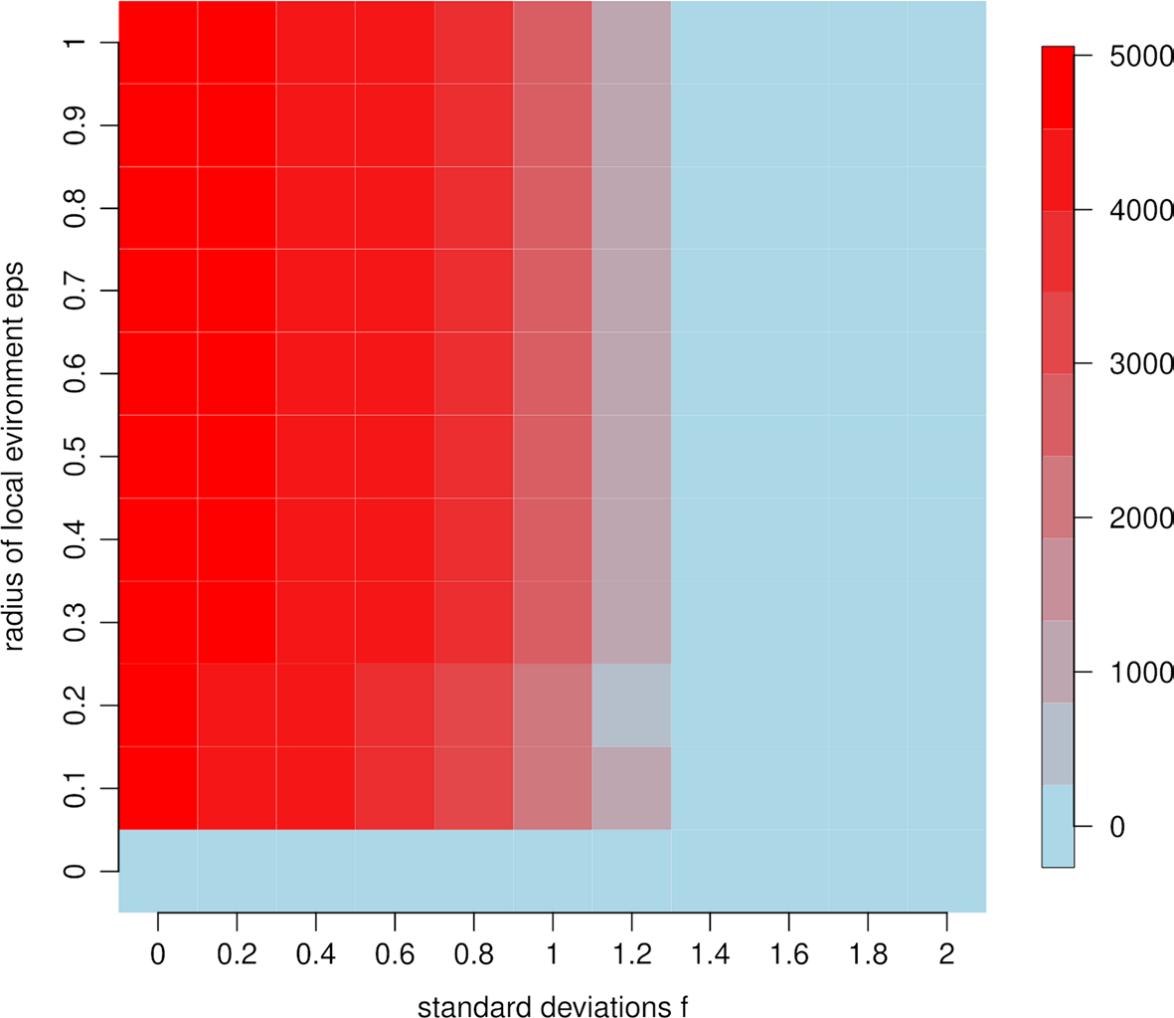
Omicron variant. Heatmap showing the number of outliers (from low, depicted in light blue, to high, depicted in red) as a function of the radius of the local environment eps and the number of standard deviations f

Before applying the approach of “Outlier detection” section, we calibrate the outlier detection on the omicron data as outlined in section “Calibration”. Figure 2 shows the two dimensional elbow plot of the number of flagged outliers as a function of both the radius of the local environment eps and the parameter f. We indeed observe a distinct shape of the decrease in the number of outliers as the parameter f increases, with a sharp decrease at around f = 1.2, after which the plot levels off. Interestingly, the algorithm is rather insensitive to the choice of the local environment eps, apart from the case eps = 0. We repeated the calibration for the other variants as well. Interestingly, the parameters f = 1.2 and eps = 1e-1 emerge as consistent choices for all variants. Therefore, we use f = 1.2 and eps = 1e-1 in the remainder of the section.

**Fig. 3:**
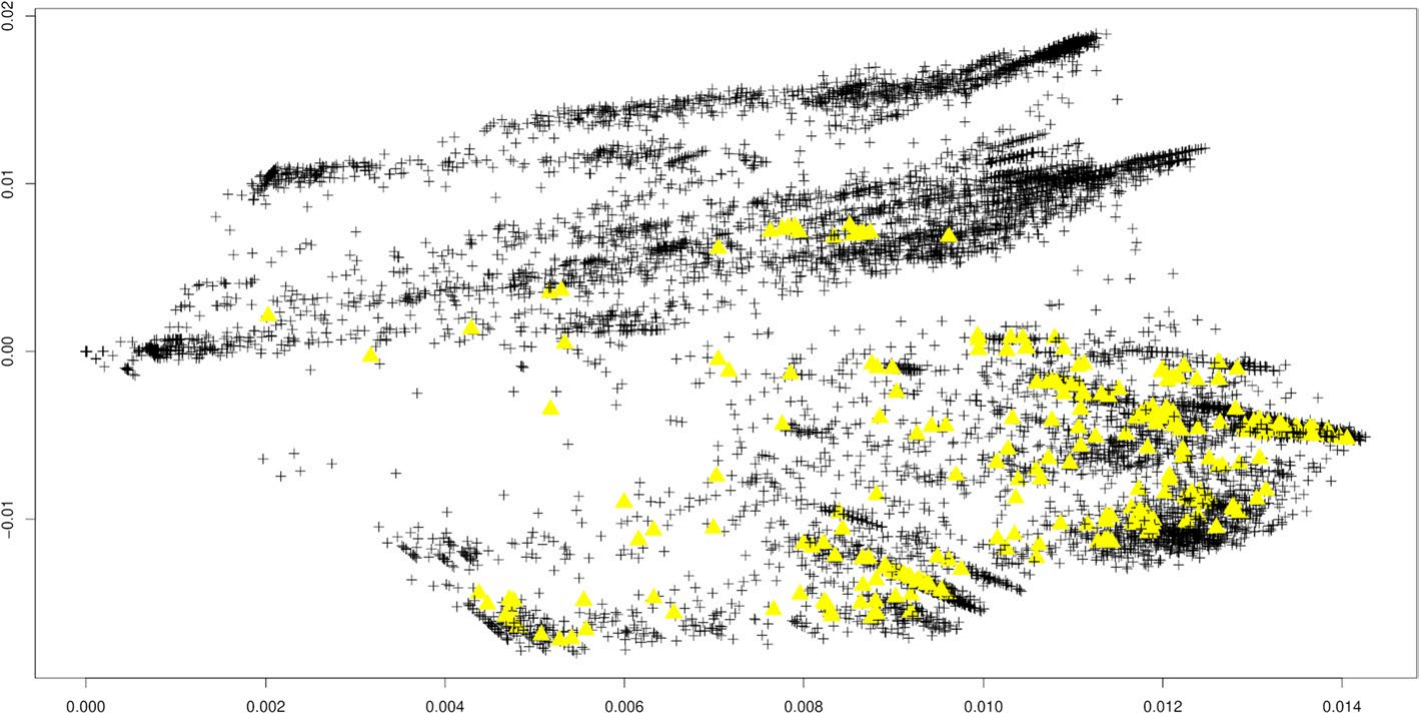
Omicron variant (see Table 4). First two principal components of the Jaccard matrix with subsequent local outlier detection approach. Parameters eps = 1e-2 (the neighborhood radius) and f = 1.5 (the multiplier for the standard deviations). Outliers depicted as yellow triangles

After calibration, we aim to identify outliers using the local detection approach of “Outlier detection” section. Figure 3 shows the same principal components as Fig. 1 for the omicron variant, though this time without any coloring by timestamp. Instead, all points in yellow have the property that they pass the local outlier criterion of “Outlier detection” section, meaning that they are outliers in a local epsilon environment centered around them, subject to the calibration of “Calibration” section.

Interestingly, using the same calibration, a number of other sequences not belonging to the omicron strain are flagged in Fig. 3. These belong to the delta variant of the SARS-CoV-2 virus. In what way these samples differ from the other delta variant samples in Fig. 3 remains an important question of future work.

**Fig. 4:**
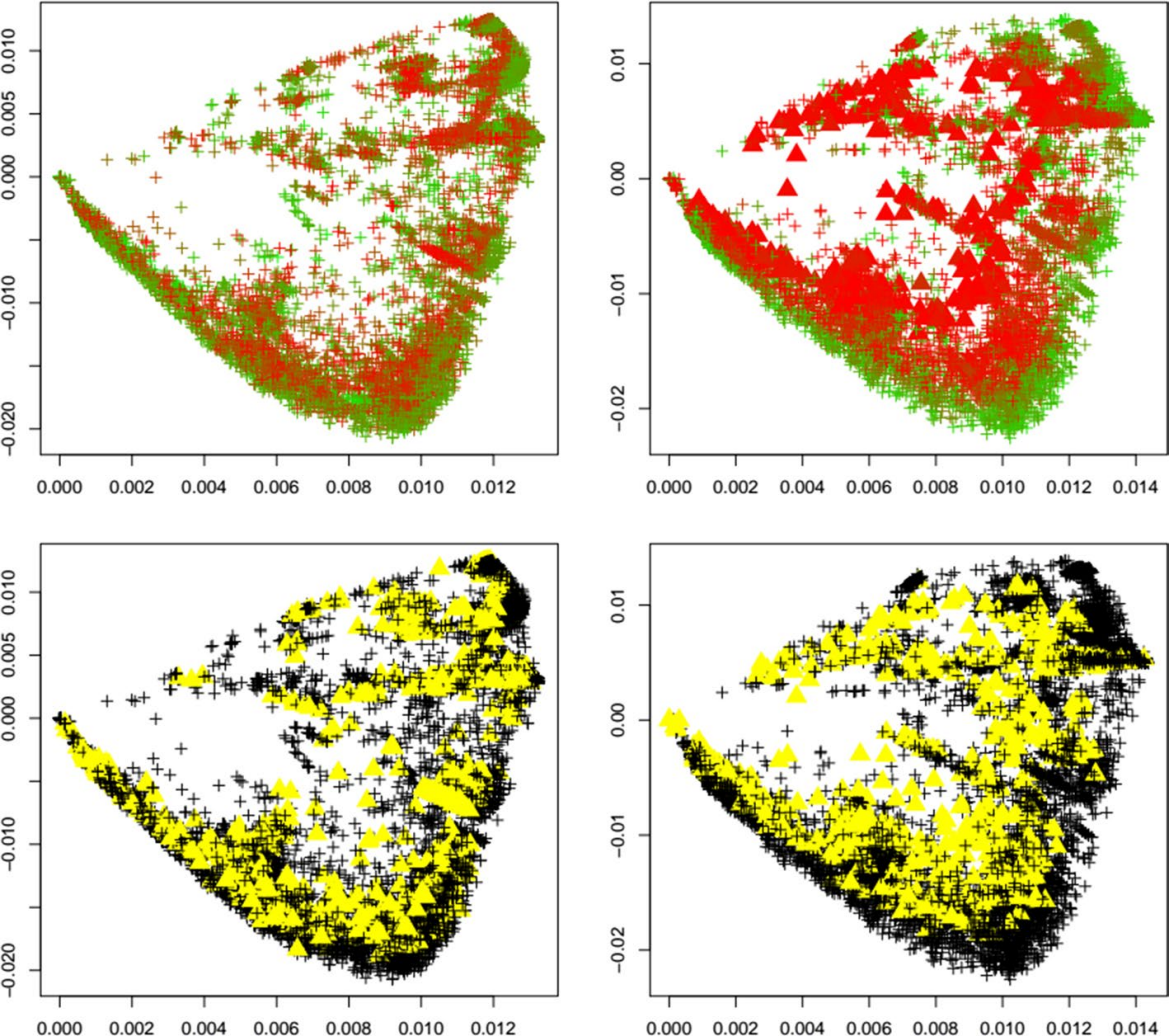
Alpha variant. First two principal components of the Jaccard matrix for the alpha variant before (top left, see Table 2) and after (top right, see Table 4) the emergence of the alpha variant, where sequences of the alpha variant (see Table 3) are highlighted as triangles. Color scheme as in Fig. 1. Local outlier detection applied before (bottom left) and after (bottom right) the emergence of the alpha variant, with outliers depicted as yellow triangles

**Fig. 5:**
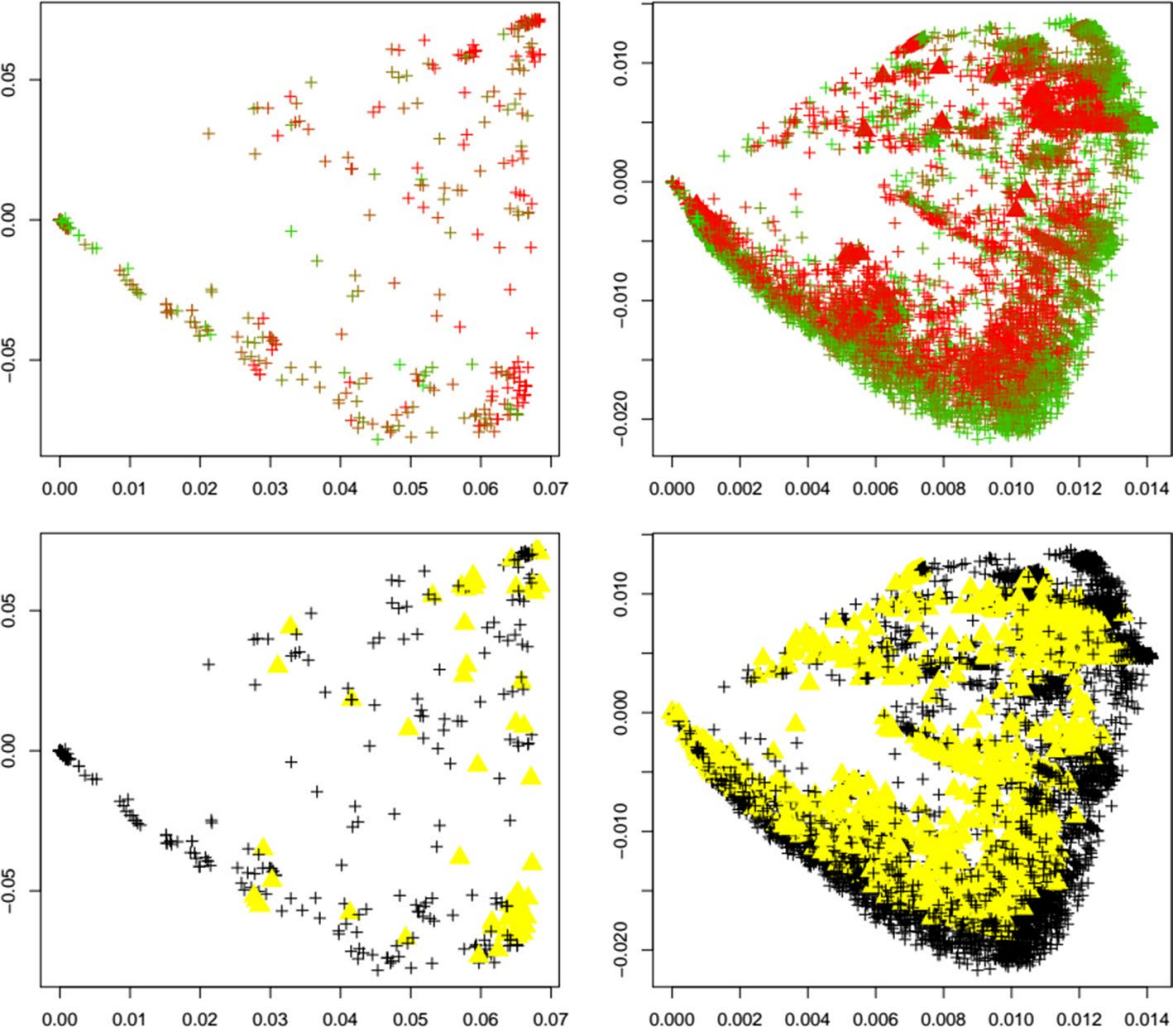
Beta variant. First two principal components of the Jaccard matrix for the beta variant before (top left, see Table 2) and after (top right, see Table 4) the emergence of the beta variant, where sequences of the beta variant (see Table 3) are highlighted as triangles. Color scheme as in Fig. 1. Local outlier detection applied before (bottom left) and after (bottom right) the emergence of the beta variant, with outliers depicted as yellow triangles

**Fig. 6:**
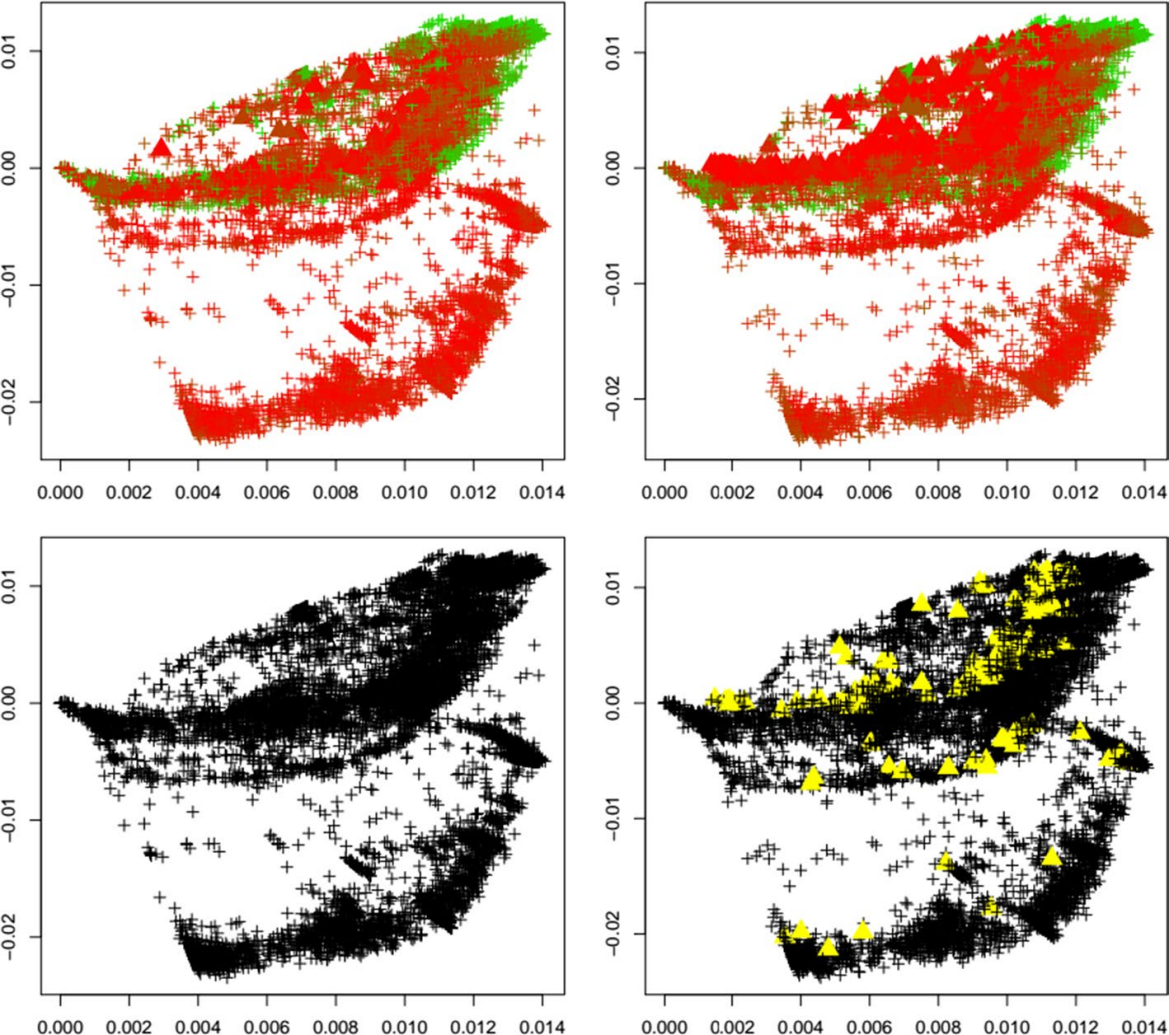
Delta variant. First two principal components of the Jaccard matrix for the delta variant before (top left, see Table 2) and after (top right, see Table 4) the emergence of the delta variant, where sequences of the delta variant (see Table 3) are highlighted as triangles. Color scheme as in Fig. 1. Local outlier detection applied before (bottom left) and after (bottom right) the emergence of the delta variant, with outliers depicted as yellow triangles

**Fig. 7:**
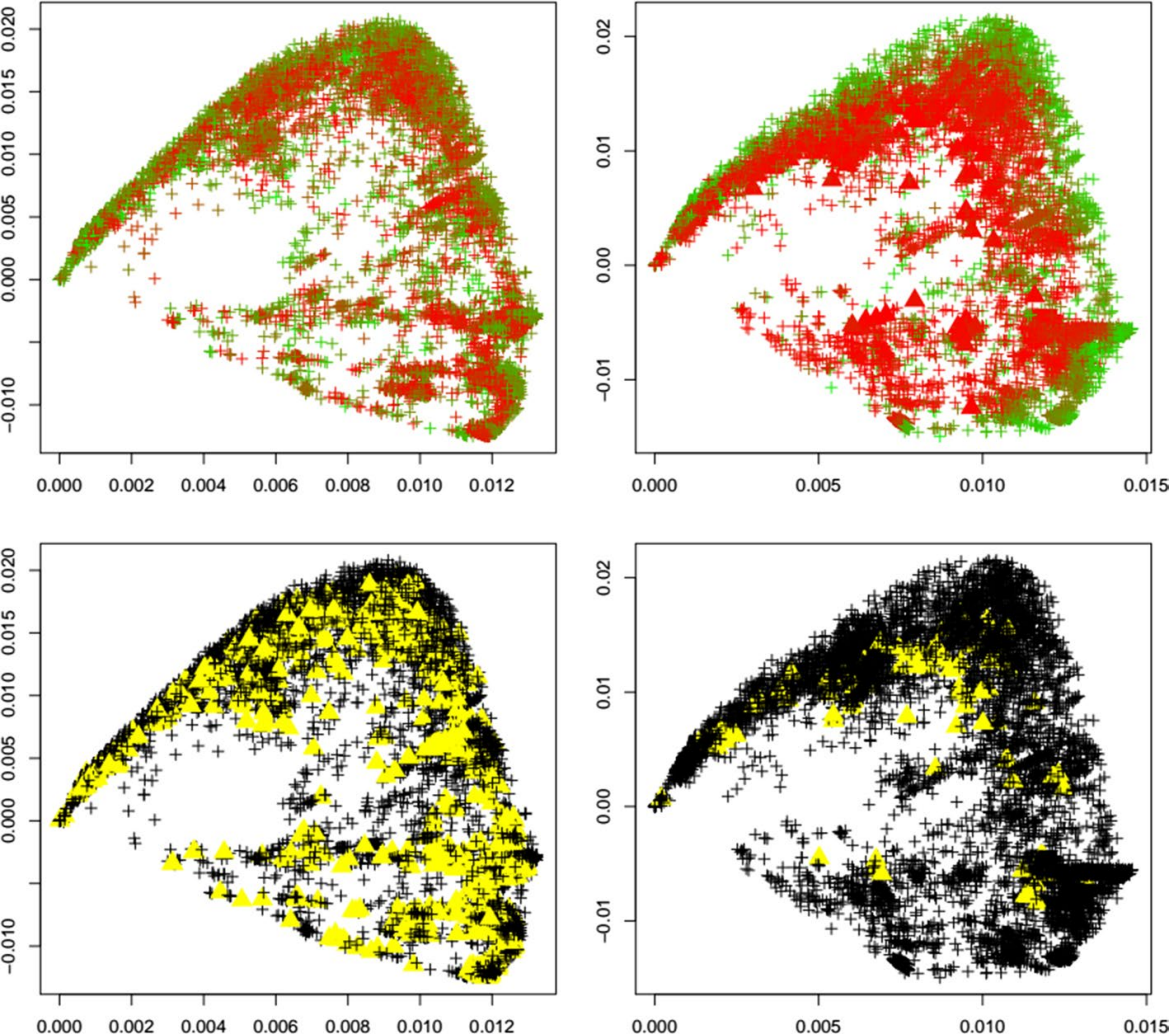
Gamma variant. First two principal components of the Jaccard matrix for the gamma variant before (top left, see Table 2) and after (top right, see Table 4) the emergence of the gamma variant, where sequences of the gamma variant (see Table 3) are highlighted as triangles. Color scheme as in Fig. 1. Local outlier detection applied before (bottom left) and after (bottom right) the emergence of the gamma variant, with outliers depicted as yellow triangles

**Fig. 8:**
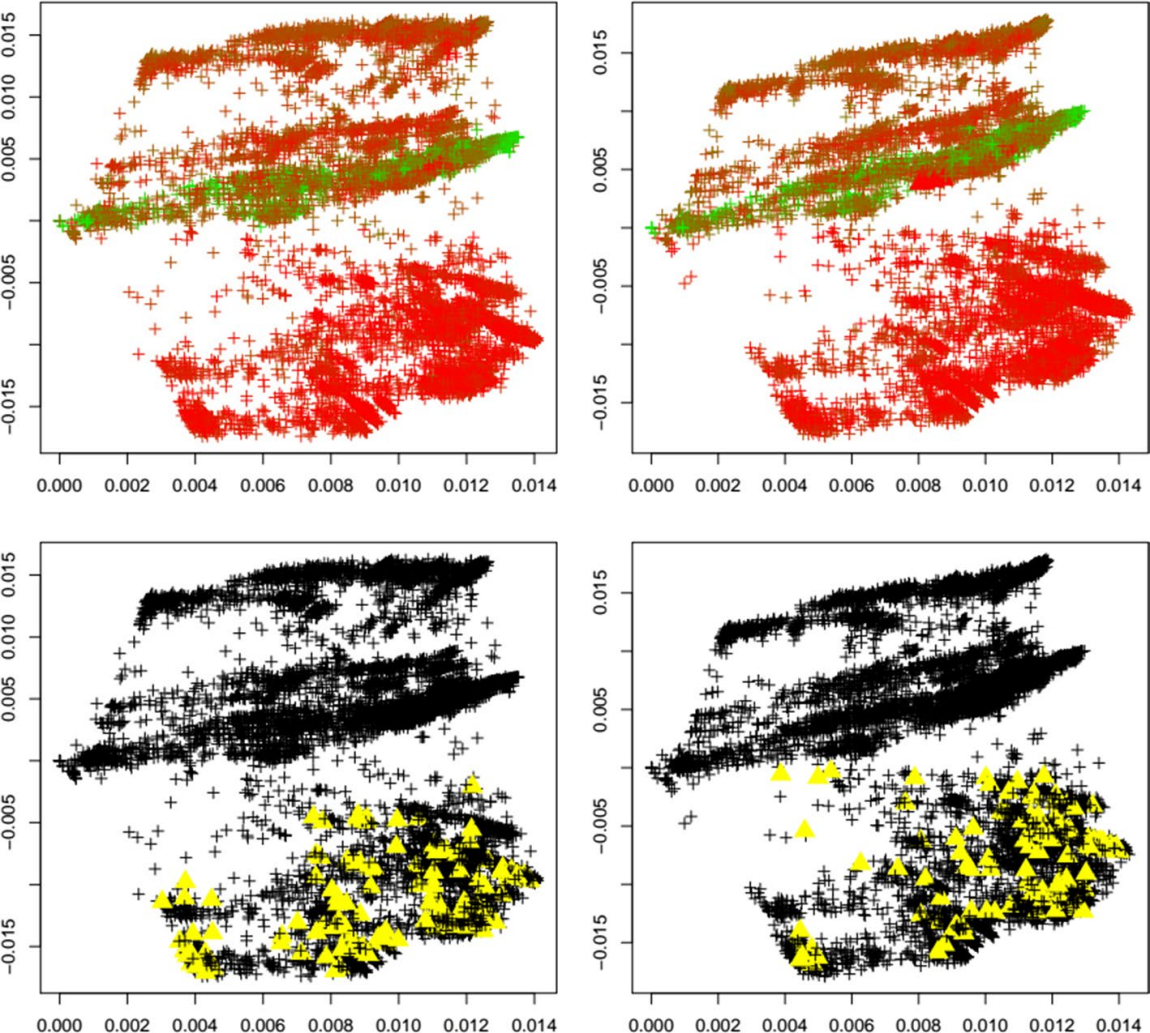
GH variant. First two principal components of the Jaccard matrix for the GH variant before (top left, see Table 2) and after (top right, see Table 4) the emergence of the GH variant, where sequences of the GH variant (see Table 3) are highlighted as triangles. Color scheme as in Fig. 1. Local outlier detection applied before (bottom left) and after (bottom right) the emergence of the GH variant, with outliers depicted as yellow triangles

**Fig. 9:**
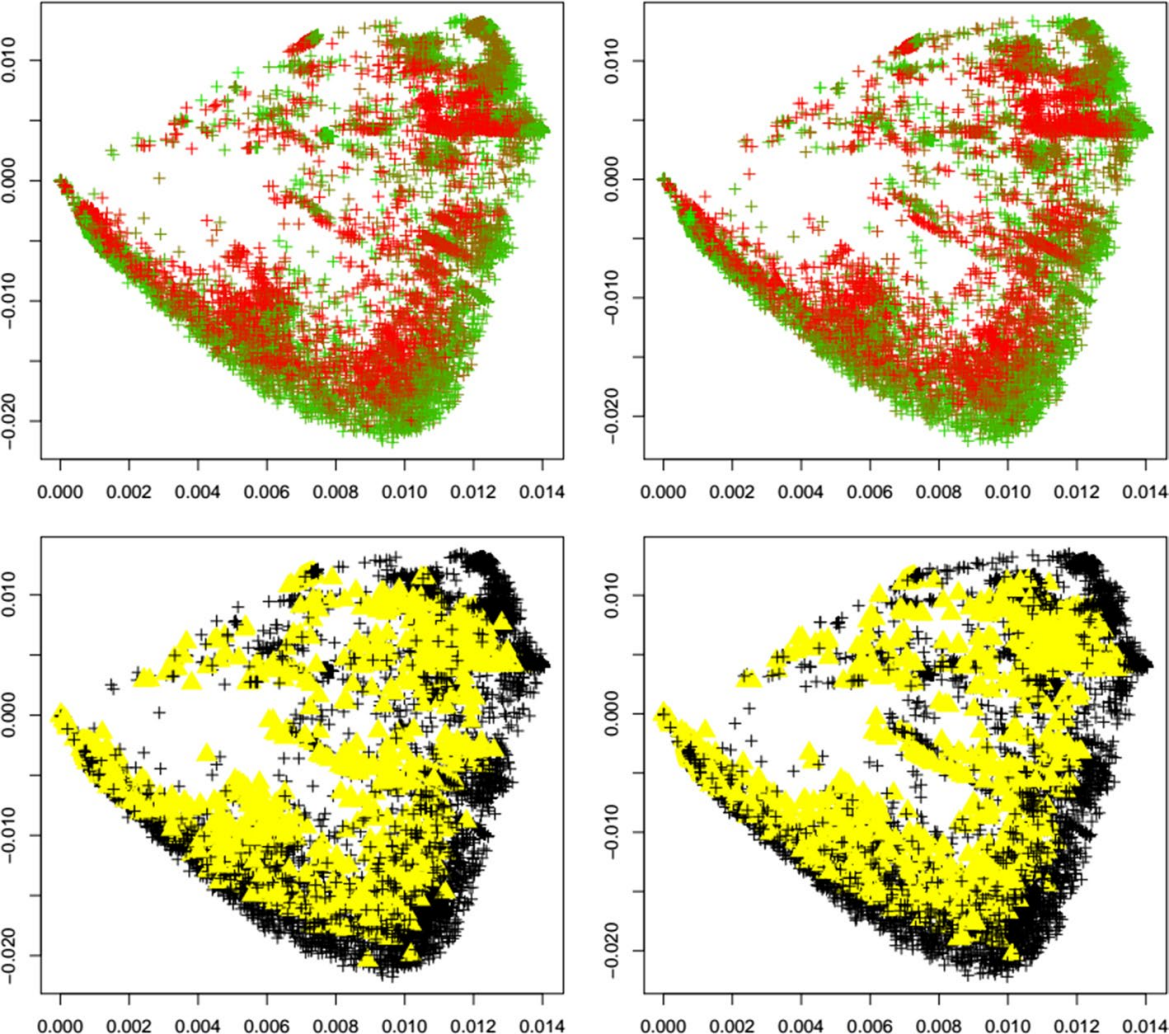
Lambda variant. First two principal components of the Jaccard matrix for the lambda variant before (top left, see Table 2) and after (top right, see Table 4) the emergence of the lambda variant, where sequences of the lambda variant (see Table 3) are highlighted as triangles. Color scheme as in Fig. 1. Local outlier detection applied before (bottom left) and after (bottom right) the emergence of the lambda variant, with outliers depicted as yellow triangles

**Fig. 10:**
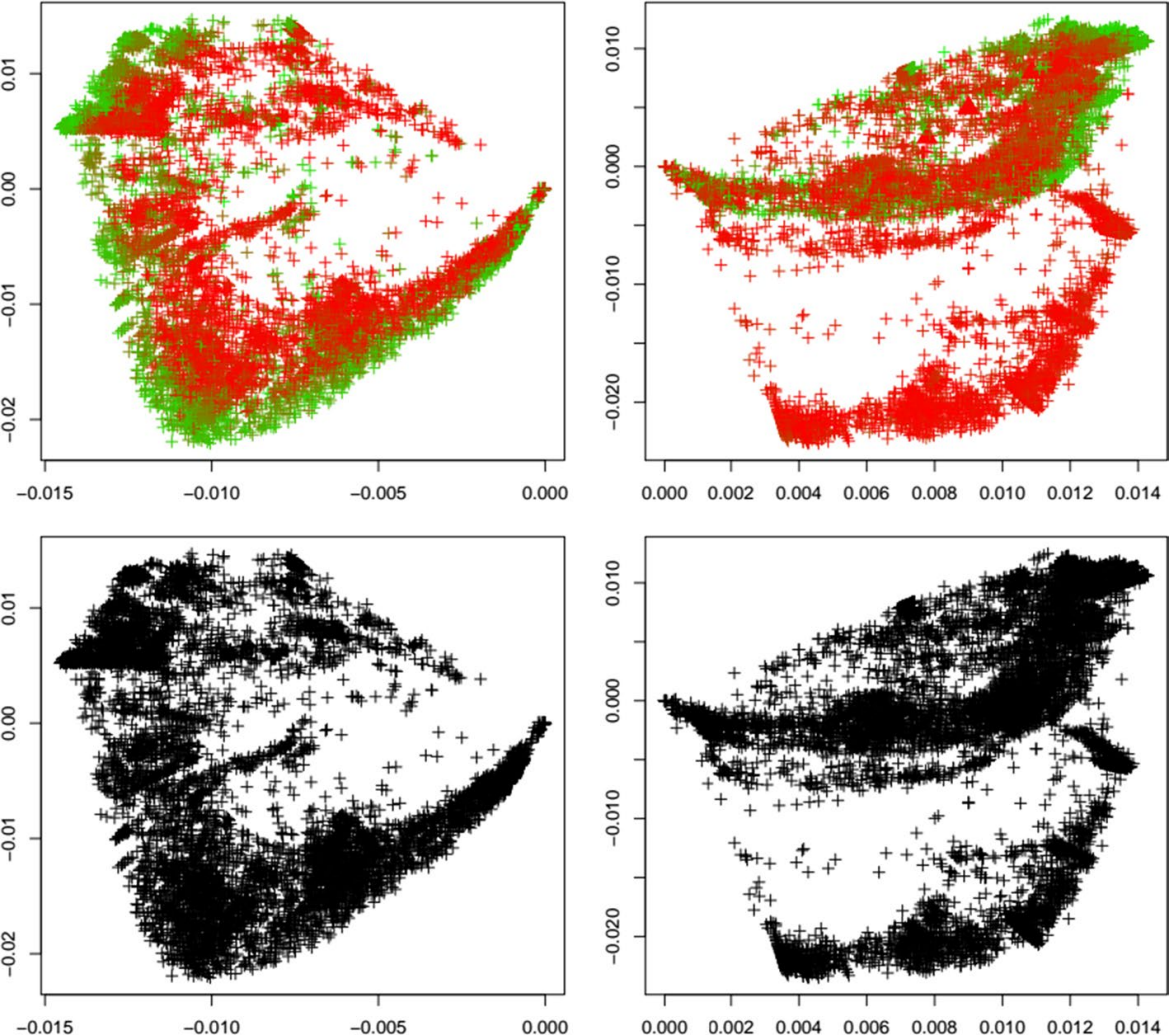
Mu variant. First two principal components of the Jaccard matrix for the mu variant before (top left, see Table 2) and after (top right, see Table 4) the emergence of the mu variant, where sequences of the mu variant (see Table 3) are highlighted as triangles. Color scheme as in Fig. 1. Local outlier detection applied before (bottom left) and after (bottom right) the emergence of the mu variant, with outliers depicted as yellow triangles

**Fig. 11:**
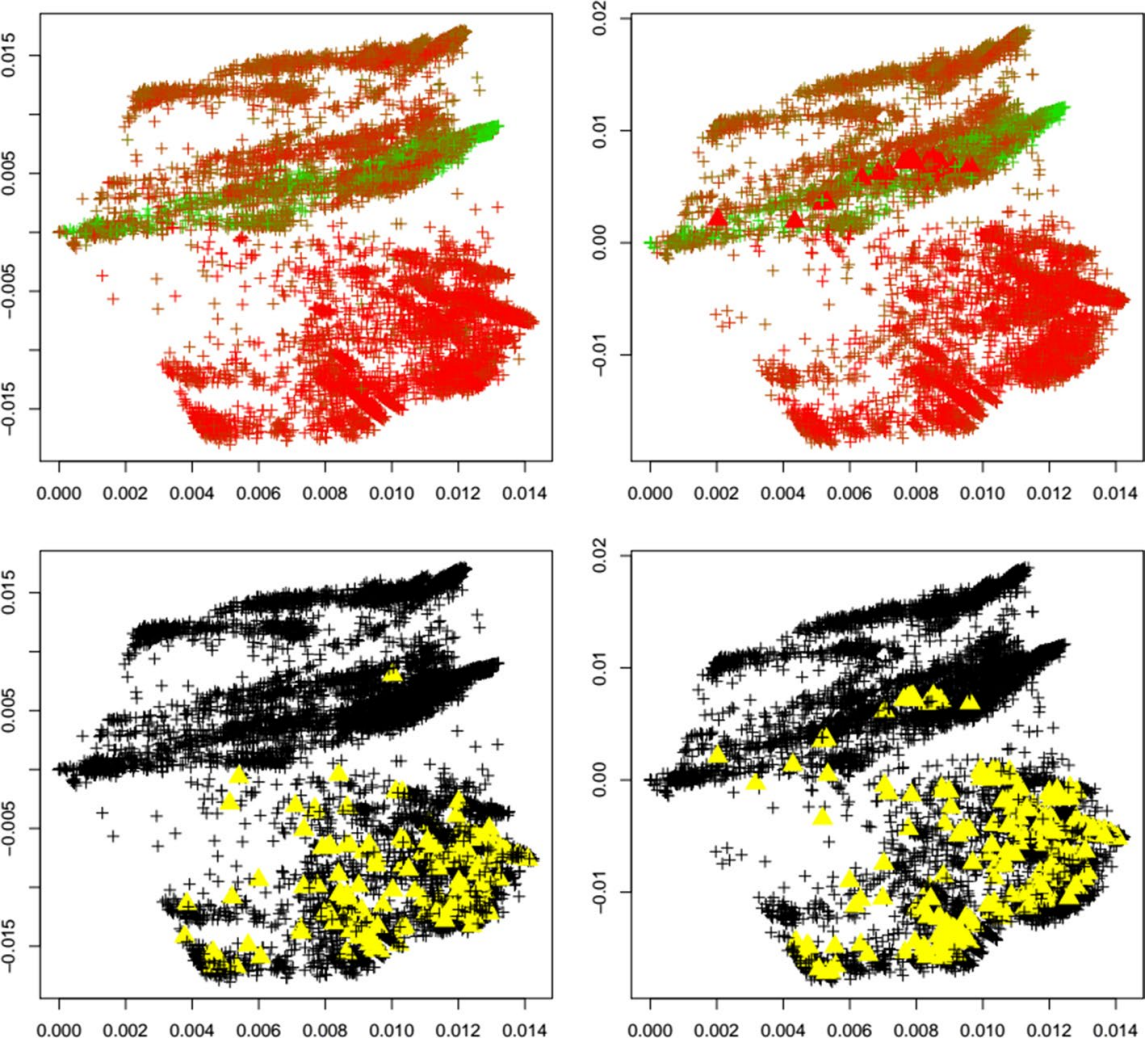
Omicron variant. First two principal components of the Jaccard matrix for the omicron variant before (top left, see Table 2) and after (top right, see Table 4) the emergence of the omicron variant, where sequences of the omicron variant (see Table 3) are highlighted as triangles. Color scheme as in Fig. 1. Local outlier detection applied before (bottom left) and after (bottom right) the emergence of the omicron variant, with outliers depicted as yellow triangles

Next, we investigate the behavior of the outlier detection upon the emergence of a new variant. We are especially interested if an increase in outliers can be detected upon the emergence of a new variant. To this end, for each variant under investigation (alpha, beta, delta, gamma, GH, lambda, mu, omicron), we apply the same calibrated outlier detected to first the reference dataset before the emergence of each variant, and after the emergence of each variant. Figures 4, 5, 6, 7, 8, 9, 10 and 11 show results for all eight variants (alpha, beta, delta, gamma, GH, lambda, mu, omicron). The left column always corresponds to the time period before the emergence of each variant, and the right column corresponds to the time period after the emergence of each variant. The top plots show the first two principal components with highlighted sequences for each variant under consideration, the bottom plots show the local outliers as yellow triangles. We observe that for the beta, delta, GH, and omicron variants the number of detected outliers considerably increases after the emergence of the variant. For the other variants, the change in the number of outliers is less pronounced. For the gamma variant, the number of detected outliers considerably decreases after the emergence of the variant.

To concretize results, Table 1 summarizes the total number of detected outliers, the number of detected genomes per variant, and the number of genomes for each variant that is included in the dataset (and that can possibly be detected). We observe that for the common variants beta, delta, GH, and omicron, the detection of the emergence of a new strain is possible. Clearly the biological importance of a new variant cannot be assessed via outlier detection, but the proposed method would have been able to flag these strains as variants of interest.

Interestingly, Table 1 shows that the number of outliers before emergence of a variant varies widely among variants. This is due to the fact that the reference datasets are independently subsampled from GISAID in order to match the timepoint T_1_ at which each variant occurs first. With our results we aim to demonstrate that a surge in outliers can happen upon emergence of a variant, meaning that the (relative) difference in the number of outliers is of interest and could be indicative of a change in the dynamics of the pandemic.

It is noteworthy to point out that in the case of Fig. 10, the plot of the first two principal components changes before and after the emergence of a variant. This is attributed to how eigenvectors (principal components) change when perturbing a matrix (for instance, [17] provides a bound on the angle of the perturbed eigenvector). Therefore, adding more data from GISAID to the computation of the Hamming matrix and the subsequent computation of the Jaccard matrix can change the Jaccard matrix and its eigenvectors.

**Fig. 12:**
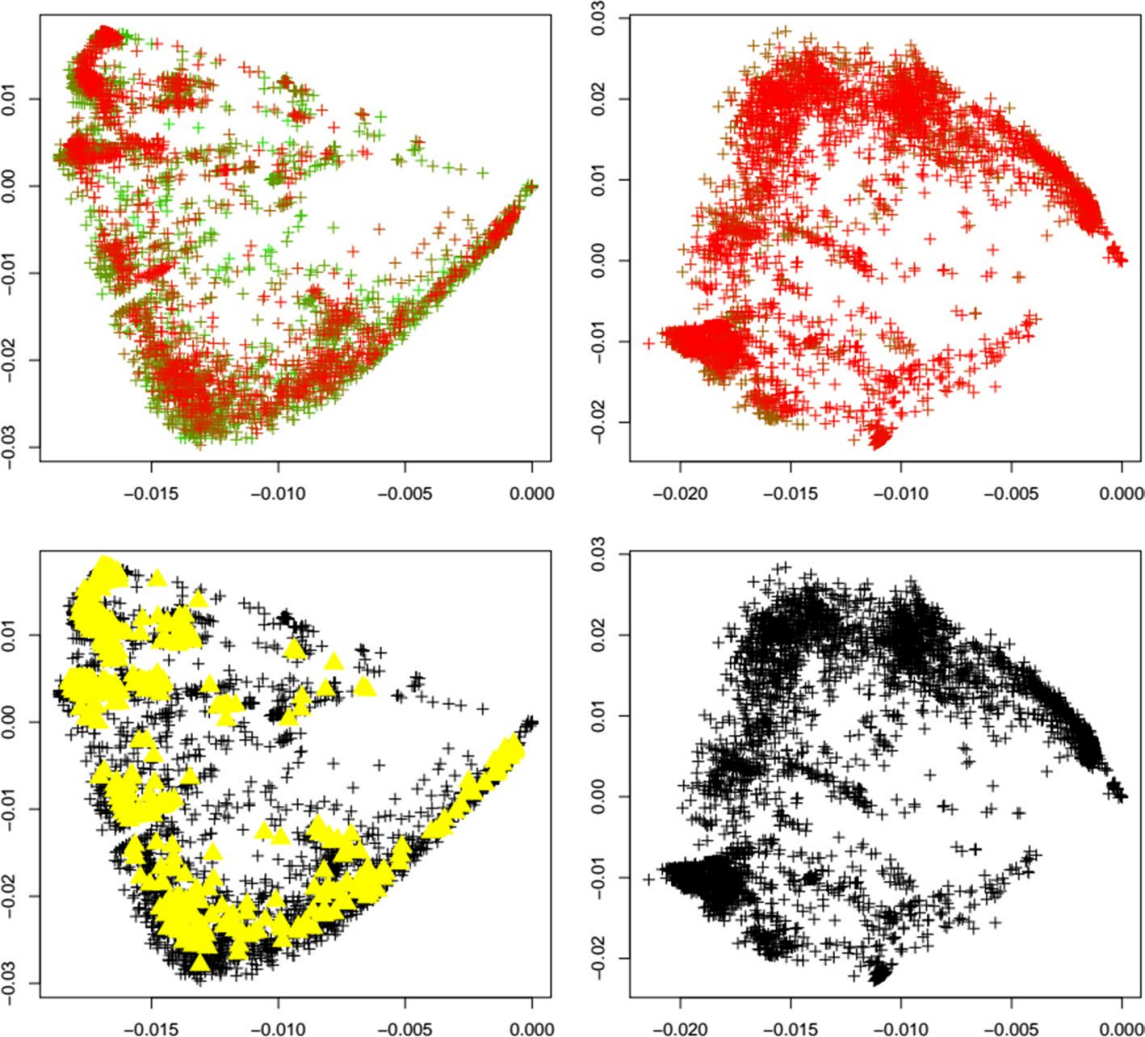
Test scenario without the emergence of a new variant. The data for this test consists of the first 5000 sequences of the reference dataset of the alpha variant (that is, before emergence of alpha) in sorted order of their timestamps (top left), while the right column depicts the second 5000 sequences before emergence of the alpha variant in sorted order of their timestamps (top right). Local outlier detection applied to the first half (bottom left) and second half (bottom right) of the dataset. Notice that repeated outliers are not shown any more in the bottom right plot

Finally, we also consider a control case in which no new variant occurs. Figure 12 shows an example of this scenario using the alpha variant. To prepare Fig. 12, we divided the reference dataset for the alpha variant (see Table 2) into two parts. The first contains the first 5000 sequences in sorted order of their timestamps, while the second part contains the later 5000 sequences. As before, we observe a certain number of outliers in the first dataset (Fig. 12, bottom left). In contrast to the other figures, sequences highlighted in Fig. 12 (bottom left) are not highlighted again in Fig. 12 (bottom right), confirming in this example that a surge would not be detected at this point in time.

## Discussion

In this work, we demonstrate that nucleotide sequences of common virus strains/variants can be identified solely based on a statistical outlier criterion in real time. To this end, we prepare two reference datasets, one before and one after the emergence of eight common SARS-CoV-2 variants (alpha, beta, delta, gamma, GH, lambda, mu, omicron) available on the GISAID database, and apply an outlier detection method to those datasets.

Using the proposed local outlier detection approach, we can identify genomes belonging to the beta, delta, GH, and omicron strain upon emergence of these variants. However, this detection comes at the cost of a larger number of false positives. The nature of those other nucleotide sequences that pass our outlier criteria, and in what way they differ from other sequences of the most common SARS-CoV-2 variants, is an important direction of ongoing research.

The large number of false positives we observe when applying outlier detection to nucleotide sequences can pose a problem for the task of accurately highlighting newly emerging sequences. The primary aim of this proposed methodology is for use as an online screening tool, or warning system, to detect the emergence of a new variant through an increase in outliers. Additional work would be required to confirm which outliers are newly emerging variants of concern.

In our study we aim to demonstrate the usefulness of the proposed methodology for prediction. However, not all mathematical models are useful prediction tools. Various prediction models have been proposed since the start of the pandemic, with various success. For instance, some models forecasted that SARS-CoV-2 would not develop any variants with distinct pathologies [18], while others concluded based on hidden Markov models that certain variants with deleterious mutations go extinct [19]. A comprehensive and retrospect assessment of the accuracy of (non-pharmacological intervention) models for the case of Sweden can be found in [20], where the authors conclude that some models significantly overestimated the virus spread.

Importantly, this research shows that outlier detection might be a useful tool to identify emerging variants in real time as the pandemic progresses, using machine learning techniques and purely statistical methods only.

An important direction of further work addresses the question of whether certain sites/loci on the SARS-CoV-2 genome are more predictive for a certain outcome than others. For instance, certain high frequency (hot spot) mutation sites are known for the coronavirus family which result in different pathologies, such as seen in the MERS-CoV nsp3 protein [21]. Similarly, future work could look into the more stable low frequency (cold spot) mutation sites, since those potentially allow for a more robust characterization of strains or new variants.

## Supplementary Information

**Additional file 1.** Lists of GISAID IDs for the two reference datasets (simulating the time before the emergence of a new variant and the onset of a new variant) for each variant under consideration in the article (alpha, beta, delta, gamma, GH, lambda, mu, omicron).

## Data Availability

The data that support the findings of this study are publicly available in the GISAID database [1, 2], see https://gisaid.org/. Additionally, the supplementary material of this manuscript contains, for each variant under consideration (alpha, beta, delta, gamma, GH, lambda, mu, omicron), lists of IDs for the two reference datasets (simulating the time before the emergence of a new variant and the onset of a new variant).
